# Correction: A nanomagnetic triazole-based Schiff-base complex of palladium(0) as an efficient heterogeneous catalyst for the Mizoroki–Heck C–C cross-coupling reaction under green conditions

**DOI:** 10.1039/d5na90046h

**Published:** 2025-07-17

**Authors:** Yassin T. H. Mehdar, Fatimah Mohammed Alshamsan, Asma Ahmad Nashawi, Hussein Eledum, Ahmed Mohajja Alshammari, Jawza A. Almutairi

**Affiliations:** a Department of Chemistry, Taibah University P. O. Box 30002 Al-Madinah Al-Munawarah, 14177 Kingdom of Saudi Arabia yassinthmehdar@gmail.com ymehdar@taibahu.edu.sa; b Department of Food Sciences and Nutrition, College of Food and Agricultural Sciences, King Saud University P. O. Box 2460 Riyadh 11451 Kingdom of Saudi Arabia Falshamsan@ksu.edu.sa; c Department Faculty of Pharmacy, University King Abdulaziz University Jeddah Kingdom of Saudi Arabia; d Department of Statistics, University of Tabuk Tabuk Kingdom of Saudi Arabia; e Department of Biology, College of Science, University of Ha'il P. O. Box 2440 Ha'il Kingdom of Saudi Arabia; f College of Pharmacy, Pharmaceutical Science, Princess Nourah Bint Abdulrahman University Riyadh Kingdom of Saudi Arabia Jawza.a.almutairi@gmail.com

## Abstract

Correction for ‘A nanomagnetic triazole-based Schiff-base complex of palladium(0) as an efficient heterogeneous catalyst for the Mizoroki–Heck C–C cross-coupling reaction under green conditions’ by Yassin T. H. Mehdar *et al.*, *Nanoscale Adv.*, 2025, https://doi.org/10.1039/d5na00364d.

The authors regret that some of the information regarding the affiliations of the authors has been displayed incorrectly in the original publication. The following shows the correct details for affiliations ‘*a*’ and ‘*f*’.

Affiliation ‘*a*’: where the spelling of ‘Al-Munawarah’ has been corrected:

^*a*^Department of Chemistry, Taibah University, P. O. Box 30002, Al-Madinah Al-Munawarah, 14177, Saudi Arabia. E-mail: yassinthmehdar@gmail.com; ymehdar@taibahu.edu.sa

Affiliation ‘*f*’: where the full title of the country ‘Kingdom of Saudi Arabia’ has been used:

^*f*^College of Pharmacy, Pharmaceutical Science, Princess Nourah Bint Abdulrahman University, Riyadh, Kingdom of Saudi Arabia. E-mail: Jawza.a.almutairi@gmail.com

The Royal Society of Chemistry apologises for these errors and any consequent inconvenience to authors and readers.

